# Metabolic Signaling in the Tumor Microenvironment

**DOI:** 10.3390/cancers17010155

**Published:** 2025-01-06

**Authors:** Ryan Clay, Kunyang Li, Lingtao Jin

**Affiliations:** Department of Molecular Medicine, University of Texas Health Science Center at San Antonio, San Antonio, TX 78229, USA; clayr@livemail.uthscsa.edu (R.C.); lik3@uthscsa.edu (K.L.)

**Keywords:** cancer metabolism, tumor microenvironment, immune response, oncometabolite

## Abstract

Cancer cells must reprogram their metabolism to sustain rapid growth. This is accomplished in part by switching to aerobic glycolysis, uncoupling glucose from mitochondrial metabolism, and performing anaplerosis via alternative carbon sources to replenish intermediates of the tricarboxylic acid (TCA) cycle and sustain oxidative phosphorylation (OXPHOS). While this metabolic program produces adequate biosynthetic intermediates, reducing agents, ATP, and epigenetic remodeling cofactors necessary to sustain growth, it also produces large amounts of byproducts that can generate a hostile tumor microenvironment (TME) characterized by low pH, redox stress, and poor oxygenation. In recent years, the focus of cancer metabolic research has shifted from the regulation and utilization of cancer cell-intrinsic pathways to studying how the metabolic landscape of the tumor affects the anti-tumor immune response. Recent discoveries point to the role that secreted metabolites within the TME play in crosstalk between tumor cell types to promote tumorigenesis and hinder the anti-tumor immune response. In this review, we will explore how crosstalk between metabolites of cancer cells, immune cells, and stromal cells drives tumorigenesis and what effects the competition for resources and metabolic crosstalk has on immune cell function.

## Simple Summary

Metabolites play a critical role within the tumor microenvironment (TME) by imposing changes on cell signaling, gene expression, and metabolic reprogramming of several tumor cell types. The TME has been found to impact the efficacy of emerging immunotherapies, and current work in the cancer metabolism space is focused on targeting the metabolic pathways that promote tumorigenesis and chemoresistance. This review summarizes recent discoveries in tumor metabolism and the impact metabolites have on altering cell signaling, preventing an effective immune response, and promoting tumor growth.

## 1. Introduction

Cancer cells exhibit altered metabolism compared to normal cell types. The first example of this was established by Otto Warburg in 1956, who showed that cancer cells increase their rate of glucose uptake and convert glycolytic pyruvate into lactate in the presence of oxygen [1,2]. In the decades since, this type of metabolic plasticity has also been observed for several other nutrient pathways, including glutamine [3,4,5] and other amino acids [6,7,8,9], lactate [10], acetate [11], and lipids [12]. It is now understood that many highly proliferative cell types perform metabolic reprogramming to drive rapid growth and to adapt to a dynamic extracellular environment. This includes immune cells and the many cell types of the tumor, all of which have distinct metabolic programs, requirement for nutrients, and ability to adapt to starvation and hypoxia [13,14]. Just as each of these cell types serve a distinct purpose in tumorigenesis and cancer progression, they also have different metabolic requirements.

## 2. Oncogenic Cell Signaling Drives Metabolic Reprogramming

The dysregulation of cancer metabolism occurs early in oncogenesis as a result of oncogenic signaling pathways, the loss of tumor suppressors, and epigenetic remodeling. Even though the initial cancer cells may be influenced by dozens of possible driver mutations, these aberrant signaling and gene expression pathways often converge on one or several of the same downstream factors that regulate cellular metabolism, meaning tumors with diverse genetic backgrounds will often modify their metabolic programming in similar ways. Here, we give a brief overview of some of the most commonly affected signaling networks.

### 2.1. MYC

MYC is a master regulator of cellular metabolism [15]. The expression of the MYC oncogene family is amplified in over 70% of cancers [15]. MYC is involved in the regulation of practically every metabolic pathway in the cell, including the induction of aerobic glycolysis, glutaminolysis, amino acid metabolism, lipid metabolism, nucleotide metabolism, polyamine synthesis, etc. [15]. In turn, MYC is also regulated by metabolic signals in several ways. For example, nutrient starvation can lead to the activation of one of several signaling pathways that results in miR-145- and miR-34b/c-mediated MYC mRNA degradation [15,16,17,18]. Conversely, nutrient availability enhances MYC mRNA expression through several mechanisms, such as the nutrient-sensing pathway mTORC1 or the GCN2–ATF4 cell stress pathway [15,19,20,21,22,23] and epigenetically through lncRNAs GLCC1, FILNC1, and PDIA3P [24,25,26]. Pathways for protein-level metabolic regulation of MYC include GSK3b-mediated proteasomal degradation or calpain protease degradation upon glucose starvation, to name a few [27,28,29].

### 2.2. HIF

Hypoxia inducible factors (HIFs) are dimeric transcription factors that are the master regulators of the hypoxia response [30]. HIFs comprise three alpha (HIF-1a/2a/3a) and one beta (HIF-1b) isoforms that increase the expression of dozens of target genes that help the cell adapt to hypoxia [31]. They do this by increasing anaerobic glycolysis and glutaminolysis, decreasing mitochondrial metabolism, and synthesizing and secreting factors to increase vascularization and oxygen delivery to the tissue [31,32]. HIF-a is the regulatory subunit for HIF activity and is regulated post-translationally by its protein stability: it associates with protein complexes containing prolyl-4-hydroxylases (PHD) and the von Hippel–Lindau (VHL) tumor suppressor, which constitutively initiate its hydroxylation and ubiquitin-mediated degradation in a reaction that requires oxygen, iron (II), ascorbate and alpha-ketoglutarate (aKG) [33,34].

Canonically, upon depletion of oxygen, the protein expression of HIF-a is stabilized, allowing for its activation of downstream target genes [35]. In cancer, however, several pathways have emerged that stabilize the expression and activity of HIF-1a even in the presence of oxygen, such as through aberrant phosphoinositide 3-kinase (PI3K)/Akt signaling [36], loss of the tumor suppressor VHL [37], expression of lincRNA-p21, which binds to HIF-1a and disrupts its interaction with VHL [38], or overexpression of deubiquitinases [30,39]. Additionally, several pathways for the metabolic dysregulation of HIF-1a exist, including mutations to TCA enzymes or downregulation of mitochondrial metabolism that reduces the supply of aKG [40,41,42]. Lactate accumulation can also increase HIF-a stabilization through a mechanism by which lactate-derived pyruvate directly competes with aKG for binding of PHD, blocking the degradation of HIFs [35]. Succinate, which is a product of the PHD hydroxylation reaction, can also accumulate in cancer cells and inhibits PHD activity when its cellular concentration is high [31,40].

### 2.3. Akt and mTOR

The PI3K/Akt pathway is the most frequently activated signaling pathway in cancer [43,44]. Akt signaling regulates many aspects of metabolism and acts through several mechanisms. As a kinase cascade, the Akt pathway can directly phosphorylate numerous metabolic enzymes to directly regulate their activity [45]. For example, Akt activation can rapidly induce aerobic glycolysis by phosphorylating several glycolytic enzymes to increase glucose uptake, increase glycolytic flux, and bypass mitochondrial uptake of pyruvate in favor of lactate production [46]. Sustained Akt activation also upregulates several metabolic pathway enzymes by inducing their transcription through downstream transcription factors such as MYC, HIF1, NRF2, ATF4, SREBP, and FOXO [45]. Akt also regulates cellular metabolism through its activation of the anabolism-regulating kinase mTOR. mTOR forms two complexes—mTORC1 (acts as a downstream effector kinase) and mTORC2 (also acts as an upstream activator/regulator of Akt) [45]. Both mTOR complexes regulate various metabolic pathways, including nucleotide synthesis, PPP, amino acid metabolism including glutaminolysis and one-carbon reactions, lipid synthesis, and protein synthesis [45]. Several miRNAs and lncRNAs have been found to regulate the Akt/mTOR pathway epigenetically [47,48]. TSC1/2 is another tumor suppressor and negative regulator of mTOR that is also frequently dysfunctional in cancers [49].

### 2.4. AMPK

AMP Kinase (AMPK) is a key energy sensor and is typically regarded as a suppressor of tumorigenesis; however, it plays a critical role in nutrient adaptation in TILs [50]. AMPK senses the energy status of the cell by binding ATP, ADP, or AMP, where ADP or AMP signals a drop in cellular energy production and allosterically activates AMPK’s kinase function [50]. Multiple upstream kinases can also activate or inactivate AMPK including liver kinase B1 (LKB1) [51], calcium/calmodulin-dependent protein kinase kinase (CamKK) [52], Akt [53] and mTOR [54]. Loss of the tumor suppressor LKB1 is common in many cancers [55]. Other mechanisms of regulating AMPK activation under energy stress, such as the lncRNA NBR2, are frequently downregulated in cancers [56]. Upon activation, AMPK generally inhibits anabolic functions and promotes catabolism [50]. AMPK activation can also induce cell cycle arrest [57] and trigger cell death pathways [58,59] when activated in cancer. In immune cells, the LKB1–AMPK signaling pathway is critical to reprogramming cellular metabolism to survive in response to nutrient deprivation in the TME [60]. For example, AMPK becomes activated upon glucose starvation to upregulate glutaminolysis, increase FAO, and promote OXPHOS to maintain ATP production in T cells [60,61]. This has the effect of generating memory T cells from effector T cell populations as well as generating anti-inflammatory Treg cells [60,61]. AMPK also plays a role in regulating phosphatase activity in CD8^+^ T cells to maintain their production of IFNg and Granzyme B [62]. In macrophages, AMPK activation suppresses HIF-1a and NFkB to favor the generation of anti-tumor M1 macrophages [63,64,65]. AMPK has also been reported to inhibit MDSC expansion and recruitment through several signaling mechanisms [50].

### 2.5. RAS and EGFR

Many common driver mutations in cancer, such as RAS- and EGFR-activating mutations, activate downstream cell signaling and converge on the same metabolic pathways to drive unrestricted growth. Mutant EGFR and KRAS both indirectly regulate cancer metabolism by activating downstream signaling pathways for metabolic regulation including Akt, MYC, and mTORC1 [66,67], which in turn regulate practically every metabolic pathway in the cell. Mutant KRAS has been shown to directly upregulate the expression of several key glycolytic enzymes including glucose transporter 1 (GLUT1) and lactate dehydrogenase A (LDHA) [68], and increases the expression of enzymes to increase flux through parallel pathways such as hexosamine biosynthesis and PPP [69]. KRAS also upregulates glutamine metabolism in the same way [70], and targets related pathways such as amino acid metabolism [71] and folate metabolism [72].

## 3. Metabolites Play Crucial Roles in Intratumoral Signaling

The local availability of nutrients and conditions within the TME can impact everything from the rate of proliferation [73] to the efficacy of the anti-tumor immune response [74], and altered nutrient utilization and generation of a toxic TME occur early in tumorigenesis [75]. In addition to cell signaling changes, several miRNAs and lncRNAs have also been implicated in reprogramming metabolism in tumor cells and in the remodeling of the TME [76,77]. As the cancer cells increase their consumption of glucose, they concurrently increase their secretion of lactate to drive glycolytic flux [2]. Many cancer cells also upregulate glutamine metabolism (glutaminolysis) to replenish intermediates of the TCA cycle that are lost from the diminished contribution from glycolysis, forcing the cell to take up extracellular glutamine [78]. This means that, even from an early stage, infiltrating immune cells must compete for limited nutrients in an environment that is flooded with waste products, has a low pH due to accumulated lactate, and may already be poorly oxygenated depending on the level of vascularization. These problems are compounded as the tumor increases in size, and results in a highly heterogeneous tumor where nutrient availability, acidity, oxygenation, and immunosuppressive conditions vary across the tumor and contribute to cancer progression, metastasis, and impaired drug delivery and treatment.

**Figure 1:**
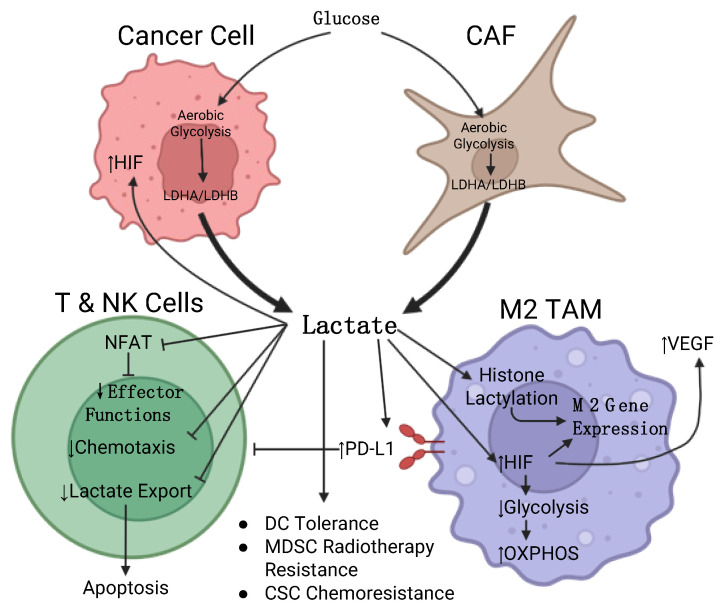
Lactate signaling in the TME: Lactate is produced in large quantities by cancer cells and CAFs and secreted into the TME. This lactate activates signaling pathways in several cell types, such as stabilizing HIF expression in the cancer cell and TAM, altering TAM gene expression to reinforce M2 polarization, triggering TAM VEGF production to promote vascularization, and inhibiting NFAT-dependent effector functions in T and NK cells. High lactate also contributes to tumor progression by directly killing anti-tumor immune cells.

It is now understood that many metabolites are either secreted into or depleted from the TME by various tumor cell types. These metabolites not only influence the conditions within the TME, but can also influence the metabolic programming, signaling, and gene expression of neighboring cells (Figure 1). In many cases, this intertumor metabolic crosstalk influences the immune cells and stromal cells within the tumor to favor a pro-tumor phenotype and diminishes the anti-tumor immune response. In this review, we will examine how metabolic features of the tumor affect signaling between cancer cells, stromal cells, and immune cells as well as how they affect immune cell activation, hamper the anti-tumor immune response, and favor the generation of immunosuppressive immune cells.

### 3.1. Glucose and Lactate

In order to fulfill their biomass and energy production requirements, rapidly dividing cells, such as cancer cells [2] and activated T cells [79], decouple glycolysis from mitochondrial ATP production in a process called aerobic glycolysis. In fact, cancer cells only come in third for highest users of glucose behind myeloid and T cells (whereas cancer cells are the biggest consumers of glutamine) [80]. Increasing the rate of glycolysis results in the rapid production of ATP from glucose, as glycolysis produces ATP about 100 times faster than OXPHOS [81]. The increase in glycolytic flux also maintains parallel biosynthetic pathways such as the Pentose Phosphate Pathway (PPP), hexosamine and amino acid synthesis [82]. This can also result in the depletion of glucose in poorly vascularized areas of the tumor [83].

In activated T cells, impedance of glycolysis by glucose limitation has several effects on effector function [84]. For example, glucose limitation blocks T cell anti-tumor function by limiting their effector cytokine production [85]. Low glucose also induces the expression of the transcription factor forkhead box P3 (FoxP3), which converts anti-tumor CD4^+^ effector T cells (Teff) cells into pro-tumor regulatory T cells (Treg) [86]. Treg cell growth is inhibited by high glucose, and their suppressive function is inhibited by glucose uptake [87]. High glycolytic flux in cancer cells also promotes recruitment of myeloid-derived suppressor cells (MDSCs) to the tumor by synthesizing and secreting G-CSF from the cancer cells [88]. In pancreatic adenocarcinoma (PDAC), chronic hyperglycemia converts PDAC stellate cells to a myofibroblast-like state that upregulates secretion of CXCL12, which activates the CXCR4 receptor on the nearby cancer cells, activating cell growth and migration through the cell proliferation pathways Erk1/2, p38, and MAP kinase [89].

While lactate has traditionally been viewed as a waste product in tumors, it is now understood that it serves several important functions for oncogenesis, including suppressing the anti-tumor immune response, being consumed as a carbon source, and acting as a signaling molecule between tumor cells [90,91]. Lactate produced by tumor cells is released into the TME where it influences the function of other cell types and generally promotes a pro-tumorigenic phenotype (Figure 1). So much lactate is produced within the tumor that lactate concentrations can be as high as 10–20 times that of normal tissues (10–30 mM versus 1.5–3 mM) [92,93]. The bulk of lactate production within tumors is the result of lactate dehydrogenase (LDHA/LDHB) from the two most populous tumor cell types—cancer cells and cancer-associated fibroblasts (CAFs) [91]. High tumoral LDHA/LDHB expression and lactate concentration are associated with cancer progression and poor prognosis in several cancers [94]. The high concentration of lactate within the TME also generates a low pH that is associated with more aggressive tumor progression and chemoresistance [95]. In regions of the tumor where glucose is extremely low, both cancer and T cells have been shown to take up and oxidize lactate and utilize it in their TCA cycles [96,97,98,99,100].

#### 3.1.1. Cancer Cells

Many cancer types have been shown to consume lactate rather than discarding it as waste, especially when glucose levels are low [96,97,98,101]. Many cancers, such as melanoma, take up exogenous lactate through monocarboxylate transporters (MCT), convert it to pyruvate via LDH, and oxidize it for energy production in the mitochondria, allowing the cell to divert more glucose into the PPP to boost NADPH production [102]. However, the fate of this lactate-derived carbon can vary greatly depending on the cancer type. For example, it was found that in HeLa (cervical cancer) and H460 (non-small cell lung cancer; NSCLC) cells, 50% of the carbon derived from extracellular lactate was used for lipid synthesis [10]. Exogenous lactate can also act in a paracrine fashion on neighboring cells of the tumor. Lactate that is imported into the cell can directly stabilize the transcription factors Hypoxia Inducible Factors 1α and 2α (HIF-1α and HIF-2α), enabling the expression of its downstream target genes [35,103]. For example, lactate-stabilized HIF-2α has been shown to activate the transcription factor c-MYC to upregulate glutaminolysis in SiHa and HeLa cells [35].

#### 3.1.2. T and NK Cells

Mechanistically, the switch from anaerobic to aerobic glycolysis in activated T cells happens in two phases. In the acute phase, which occurs over the first several hours, ligation of the T cell receptor (TCR) activates downstream tyrosine kinase signaling cascades that activate pyruvate dehydrogenase kinase (PDHK1) to inhibit mitochondrial pyruvate dehydrogenase (PDH) from importing pyruvate, thereby detaching glycolysis from mitochondrial metabolism [104]. During the later phase of T cell activation, CD28 co-stimulation activates Akt, mTOR, MYC and SREBP signaling to increase spare mitochondrial respiratory capacity [105], and to upregulate the expression of glycolytic, glutaminolytic, and fatty acid synthase (FAS) genes, which further increases the rate of flux through glycolysis, glutaminolysis, and lipid synthesis pathways [106].

Lactate disrupts the anti-tumor function of tumor-infiltrating lymphocytes (TILs) through several mechanisms. Generally, when extracellular lactate is high, the secretion of glycolytic lactate from TILs is disrupted, resulting in disruption of glycolysis [107], the fragmentation and disruption of mitochondrial function [108], and a net import of lactate into the cytoplasm and lowering of the intracellular pH [109]. Cytoplasmic lactate disrupts glycolysis and energy production by T and natural killer (NK) cells by directly inhibiting the activity of the glycolytic enzymes hexokinase (HK) and phosphofructokinase (PFK) as well as disrupting the rate of flux through the pathway through accumulation of its end product pyruvate [109,110,111]. Further, lactate concentrations of 20 mM or more have been found to directly induces apoptosis in T and NK cells [109]. Intratumoral lactate also prohibits the expression of nuclear factor of activated T cells (NFAT) family transcription factors in T and NK cells, thereby limiting their effector functions and expression of pro-inflammatory cytokines, including interferon-γ (IFNγ) [109,112]. Chemotaxis and migration of CD4^+^ and CD8^+^ T cells are disrupted by elevated tissue lactate [113]. Lactate also suppresses CD8^+^ T cell release of cytolytic perforin and granzyme in vitro [113].

#### 3.1.3. Treg Cells

Treg cells show a distinct metabolic program from Teff cells. Rather than rely on aerobic glycolysis and lactate production, high lactate in the TME induces a switch from glycolysis to OXPHOS in Treg cells [87,114]. OXPHOS is critical to maintaining Treg immunosuppressive function in low-glucose environments by allowing them to metabolize lactate; inhibition of mitochondrial oxidation of lactate and lipids disrupts their function and survival [115,116]. Tregs adapt to the tumor’s high lactate and low glucose through FoxP3 [114]. FoxP3 does this by transcriptionally repressing MYC, suppressing glycolysis, increasing the rate of OXPHOS, and reversing the reaction catalyzed by LDH to consume lactate and recycle and maintain a pool of NAD+ [114]. Lactate can also induce the expression of the immune checkpoint protein programmed cell death protein 1 (PD-1) in Treg cells through NFAT [117].

#### 3.1.4. Tumor-Associated Macrophages

Tumor-associated macrophages (TAMs) can display a range of phenotypes within the tumor, mainly categorized into M1 pro-inflammatory and anti-tumor cells, or an M2 pro-tumor, anti-inflammatory programming. TAMs initially respond to the hypoxic environment of the tumor by altering their metabolism to favor aerobic glycolysis, increased PPP for NADPH production, and amino acid and FAS, which is characteristic of the M1 TAM phenotype [118]. However, in addition to differentiation signals such as IL-4, IL-10 and IL-13, the high lactate within the TME also drives M2 polarization through several mechanisms [118]. First, lactate stabilizes and activates HIF-1α, which is required for M2 TAM gene expression and polarization [119]. HIF-1α in turn promotes a switch from glycolytic metabolism to OXPHOS via lactic acid and lipid oxidation [119]. In addition to energy production, lactate can also be utilized in the lactylation of histones that helps convert TAMs from the M1 (anti-tumor) to the M2 (pro-tumor) phenotype [120]. Lactate also inhibits the expression of ATP6V0d2, which normally degrades HIF-2α in lysosomes, thereby maintaining HIF function and sustaining M2 TAM functions [121]. Lactate can also increase the expression of the immune suppressive molecule programmed death-ligand 1 (PD-L1) on the surface of the TAM, which induces apoptosis in Teff cells [122]. Lactate-HIF-1α signaling in TAMs is crucial for promoting vascularization of the tumor: while TAMs only constitute 1–6% of the cellularity of the tumor, they were found in one study to express more vascular endothelial growth factor (VEGF) mRNA than the rest of the tumor combined [119].

#### 3.1.5. Cancer-Associated Fibroblasts

In many cancers, it has been found that CAFs respond to tumor-derived proinflammatory cytokines by producing and secreting lactate [123]. In these cells, hypoxia causes stabilization of HIF-1α and increases expression of glycolytic genes that is reinforced through epigenetic regulation [124]. In other cases, cell-to-cell contact with cancer cells also triggers CAF metabolic rewiring to favor increased glycolysis via a mechanism involving SIRT3-produced reactive oxygen species (ROS) and HIF-1α stabilization, resulting in increased expression of monocarboxylate transporter 4 (MCT4) and export of lactate [125]. Lactate secreted by cancer cells and CAFs also blocks monocyte, MDSC, and dendritic cell (DC) differentiation, activation, and antigen presentation [126,127]. CAFs also secrete several other nutrients to support tumor growth including pyruvate, ketones, lipids, nucleotides, glutamine, and other amino acids [123,128].

#### 3.1.6. Dendritic Cells

Upon activation of its toll-like receptor (TLR) by a tumor antigen, DCs quickly switch to aerobic glycolysis with lactate production and FAS via the TRAF6-PI3K-Akt, mTOR, and HIF-1a signaling pathways [129,130]. The high concentration of extracellular lactate prevents the diffusion of glycolytic pyruvate, and this accumulated lactate shifts the DC into a tolerogenic phenotype upon TLR stimulation [131].

#### 3.1.7. Myeloid-Derived Suppressor Cells

In another study, MDSCs displayed high glucose and glucose-6-phosphate, but no change in other glycolytic intermediates or lactate [132]. In pancreatic cancer, the lactate receptor GPR81 has been shown to respond to extracellular lactate by activating the MDSCs and generating resistance to radiotherapy by signaling through mTOR, HIF-1α and STAT3 [133].

#### 3.1.8. Cancer Stem Cells

Although it is unclear whether cancer stem cells (CSCs) arise from the accumulation of mutations within progenitor cells native to the host tissue or evolve from differentiated cancer cells, a few hallmarks of altered metabolism in these populations have been identified. CSCs are similar metabolically to cancer cells in that they also perform anaerobic glycolysis, converting glucose to lactate and bypassing the mitochondria even in the presence of oxygen [134]. However, CSCs are dependent on glucose for survival, and glucose starvation causes rapid depletion of CSCs [135]. CSCs also demonstrate an even higher dependence on glycolysis than cancer cells [136,137] and a relatively lower reliance on mitochondrial metabolism [135]. Glycolysis can also induce chemoresistance in some CSCs by activating the Akt pathway and inducing the expression of drug efflux pumps [135].

### 3.2. Glutamine

Glutamine is the second most abundant small molecule in the serum behind glucose and is one of the most important metabolites in the tumor. Glutamine is a nonessential amino acid that is consumed in such quantity that it becomes conditionally essential to many cancers [138]. Glutamine metabolism plays several critical roles within the cancer cell beyond providing carbon to the TCA cycle via its downstream metabolite alpha-ketoglutarate (αKG) [78]. It can also be used in reductive carboxylation reactions for lipogenesis, contributes nitrogen for nucleotide synthesis, and is required for glutathione (GSH) synthesis [78]. Glutamine also drives the methionine and urea cycles as well as the transamination and one-carbon metabolism pathways that produce half of the nonessential amino acids (NEAA) in the cell [139,140]. Glutaminolysis regulates several homeostatic pathways such as redox balance [71], mTOR signaling and autophagy [141], apoptosis [142], and ferroptosis [143]. Glutamine metabolism also influences cell signaling pathways that are important for chemoresistance including RAS [69], Hippo [144], and NRF2 [145,146]. For these reasons, glutamine metabolism is frequently upregulated in cancer cells [78], and cancer cells demonstrate the highest rate of glutamine uptake of any tumor cell type [80]. Glutamine and glucose metabolism also appear to be somehow linked in tumors: inhibiting glutamine uptake with the ASCT2 inhibitor V-9302 increases glucose uptake and utilization [147], but inhibiting global glutamine metabolism with the glutamine enzymatic inhibitor 6-diazo-5-oxo-norleucine (DON) also decreases the rate of glucose metabolism in cancer and T cells [148].

In normal physiology, glutamine is used to transport carbon and nitrogen between tissues, and it serves largely the same purpose within the tumor: CAFs produce excess glutamine from various sources including branched-chain amino acids (BCAA) and aspartate metabolism and secrete it for use by cancer cells [149]. Immune cells also require glutamine for their activation and proliferation [148]. In another study, increased glutamine consumption within tumor cells was found to promote recruitment and generation of MDSC to the tumor by stabilizing the expression of the transcription factor laryngeal adductor paralysis (LAP) and increasing expression of colony stimulating factor 3 (CSF3) [150]. In macrophages, glutaminolysis is required for M1 macrophage cytokine production, antigen presentation, and phagocytosis [151], but higher rates of glutamine metabolism supports TAM polarization into the M2 phenotype [152]. Mechanistically, it is thought that increased glutamine metabolism provides excess αKG for epigenetic remodeling enzymes, which supports the expression of M2-like anti-inflammatory programs [152]. Treg differentiation by FoxP3 is also inhibited by high rates of glutaminolysis [153]. NK cells require sustained glutamine metabolism to maintain MYC expression, which regulates the expression of glycolytic and glutamine metabolic enzymes [154]. The MYC protein has a half-life of only 30 min [155], and because glutamine supports the uptake and synthesis of other amino acids, MYC must increase glutaminolytic flux to maintain its own expression [156]. Glutaminolysis is also crucial for supporting redox and GSH metabolism in DCs and helps to maintain their activation and antigen presentation [157]. In some CSC studies, it was found that glutamine is essential to maintain OXPHOS [158]. CSCs are also better at upregulating glutaminolysis in response to glucose starvation than differentiated cancer cells [158], and inhibition of glutamine metabolism reduces expression of stem markers and sensitizes CSCs to radiation [159].

### 3.3. Tryptophan

Tryptophan is an essential amino acid (EAA) that is critical for maintaining NAD+ synthesis through the kynurenine pathway [160]. Tryptophan is critical for immune cell survival, but is often depleted from the TME by cancer cells and CAFs [161]. Cancer cells and CAFs both express the enzyme indoleamine 2,3-dioxygenase (IDO), which affects T cell function in two ways. First, it depletes the TME of tryptophan, starving TILs of this EAA, inducing GCN2 activation and mTOR inhibition, and leads to anergy and cell cycle arrest [162]. Second, IDO converts tryptophan to kynurenine, which is secreted by the cell and activates the aryl hydrocarbon receptor (AhR) in CD4^+^ T cells, which causes them to differentiate into Tregs [162,163]. Kynurenine also induces PD-1 expression on CD8^+^ T cells [164]. Together, these mechanisms suppress effector T cell function, impede DC function, and induce the differentiation of Treg cells [162,163,165].

### 3.4. Arginine

Arginine is another conditionally essential amino acid to many cancers since most do not express argininosuccinate synthetase (ASS1) and cannot perform de novo arginine biosynthesis via the urea cycle [166,167]. This renders many cancer cells dependent on extracellular arginine [168]. Arginine plays several important roles in the cancer cell including polyamine synthesis and nitric oxide (NO) production [169]. CAFs, TAMs, MDSCs and cancer cells can inhibit T cell function by depleting arginine from the TME [161,170,171,172]. In TAMs and CAFs, this increase in arginine metabolism is typically the product of TGFβ signaling and HIF-1α-induced metabolic reprogramming [170,171,173,174]. Arginine catabolism occurs through two enzymes—nitric oxide synthetase (NOS) and arginase 1 (Arg1) [175]. NOS expression is disfavored within tumors because its metabolism of arginine produces NO, which produces a more inflammatory phenotype by promoting T cell extravasation and infiltration into the tumor [175], and also inhibits the tryptophan catabolic enzyme IDO [176]. IDO expression by DCs, Tregs, and CAFs deplete arginine from the TME, which inhibits Teff cells while promoting Tregs [177]. TAMs and MDSCs also secrete Arg1 into the extracellular space to inhibit neighboring T cells in a paracrine fashion by depleting arginine from the TME [75,178,179]. By secreting polyamines into the extracellular space, M2 TAMs signal to cancer cells to increase cell division [119]. In T cells, arginine normally promotes increased OXPHOS and induces stronger activation, effector function, survival, and memory formation [180]. Arginine depletion in the TME resulting from high expression of Arg1 in other tumor cells causes inhibition of mTORC1 activity in T cells, resulting in a decrease in effector functions and an increase in memory phenotype [175,180]. In NK cells, arginine depletion reduces IFNγ production, inhibits cytotoxicity, and affects cell viability [181]. Arginine depletion can also cause immunosuppression by stimulating the expression of VISTA and CD39L1 on MDSCs [182].

### 3.5. Methionine

Methionine is required for several important processes in the cell. This includes protein synthesis, nucleotide synthesis, and by providing S-adenosylmethionine (SAM), the methylation source for DNA, histone, and proteins [183]. Many cancers are addicted to methionine [184]. Cancer cells maximize their methionine uptake by upregulating the methionine transporter SLC43A2 [185] in addition to increasing production from their endogenous methionine cycle [183]. This limits the amount of methionine that is available to T cells in the tumor, which results in lower production of SAM and the loss of dimethylation at histone H3K79me2 [185]. This alters epigenetic control and in turn blocks T cell immunity by reducing the expression of STAT5-derived cytokines [185]. Metabolic-epigenetic rewiring also occurs in M2 TAMs by increasing methionine metabolism and expression of methionine adenosyltransferase 2A (MAT2A), which reinforces M2 polarization by increasing the production of SAM and promoting downstream H3K4me3 histone methylation [186], and also contributes to methionine depletion in the TME.

### 3.6. Serine and Glycine

Serine plays an important role in one-carbon reactions and DNA synthesis and is similar to glutamine in that many cancer cells rely on both an increase in its de novo synthesis (from glucose) as well as its import from extracellular sources [187]. Serine synthesis is an offshoot from glycolysis that also produces NADH and αKG [188]. Once serine is produced, it can then be converted to glycine or vice versa in order to fuel one carbon metabolism [189], purine synthesis [190], folate synthesis [191], lipid synthesis [192], and protein translation [188]. The availability of serine in the TME can vary between cancer types. In cases where cancer cells absorb the majority of serine from the TME, they can deprive T cells of serine, which is required for T cell expansion and effector functions [193]. Teff cells require exogenous serine for expansion and utilize it in serine/glycine one-carbon reactions to support growth, energy production, and to build an anti-tumor response [193]. However, a meta-analysis of cancer tissue metabolites shows serine and glycine are two of the most upregulated metabolites in the TME, even more abundant than adenosine [194]. More investigation is necessary to delineate the roles of serine and glycine in specific cancer types.

### 3.7. Cysteine

Cysteine is an important amino acid in cancer because it contributes to protein synthesis, acts as a carbon source for the synthesis of other amino acids, and is the limiting reagent in GSH synthesis [195,196]. Synthesizing GSH from cysteine fights against oxidative cell death and contributes to tumor progression [197]. Cysteine is also an important precursor for sulfur-containing molecules like coenzyme A and biotin, and participates in several post-translational modifications [196]. Many cancer cells upregulate the cystine-glutamate antiporter xCT (a dimer of SLC7A11 and SLC3A2) to resist ferroptotic cell death, and as a result, cancer cells often deplete cysteine from the TME (xCT imports cystine, a dimer of cysteines that is quickly converted to cysteine within the reducing environment of the cytoplasm in a reaction that consumes NADPH) [198].

Naïve T cells do not express the proteins required for cystine uptake or conversion of methionine into cysteine. Rather, they rely on the secretion of cysteine from macrophages and DCs by their production and secretion of GSH into the TME, which is cleaved in the extracellular space to produce cysteine that is then taken up by the ASC neutral amino acid transporter on T cells [199,200]. Treg cells inhibit this DC-mediated extracellular GSH cycle as part of their inhibitory function against Teff function [200]. In ovarian cancer, stromal cells will also upregulate xCT in order to produce excess GSH and cysteine, which is secreted from the stromal cell to the cancer cell to convey cisplatin resistance [201]. Cancer cells and MDSCs also limit the T cell’s access to cysteine by importing as much of it as possible, thereby depleting it from the TME and limiting TIL activation and function [197].

### 3.8. Alanine

Alanine is the second most abundant amino acid in the serum, but relatively little is known about its metabolic functions in the tumor. Recently, it was discovered that PDAC cells reprogram nearby stellate cells with a catabolic phenotype, which causes them to upregulate autophagy and feed nearby PDAC cells with the excess alanine and other NEAA [202,203]. The PDAC cells, in turn, convert alanine to pyruvate and utilize it in the TCA cycle, reducing their reliance on glucose and glutamine [203]. In another example, alanine-derived αKG is required for collagen hydroxylation for the preparation of metastatic niches in breast cancer metastasis [204]. Activation of naïve T cells and reactivation of memory T cells also requires extracellular alanine [205]. T cells choose to upregulate the expression of alanine importers over synthesizing alanine from pyruvate so as not to deplete pyruvate necessary for energy production [205]. T cells instead directly imported alanine for protein synthesis [205]. Alanine deprivation also impairs T cell metabolic reprogramming upon stimulation, and these cells fail to exit quiescence as a result [205].

### 3.9. Adenosine

Adenosine is a purine nucleoside that is produced in abundance in tumors and is a major contributor to generating an immunosuppressive TME [206]. Under hypoxia, tumors as well as other tissues secrete adenine nucleotides (ATP, ADP, AMP) in response to inflammatory signals [207]. Interstitial adenosine levels can reach micromolar concentrations in tumors and are present at levels 10–20 times that of normal tissues [208]. Tumoral extracellular adenosine is produced through three main pathways. First is the classical pathway where adenosine is produced from adenine nucleotides by nucleotidases CD39 and CD73 on the surfaces of cancer cells and Tregs [209,210]. Mechanistically, extracellular ATP or ADP is converted to AMP by CD39, then CD73 converts AMP to adenosine [209]. The second pathway sees NADH converted into AMP by CD38 and CD203a, which is then converted to adenosine by CD73 [211]. Finally, another option is the hydrolysis of S-adenosylhomocysteine (SAH) by adenosylhomocysteinase (AHCY) [211]. As a result, adenosine accumulates in the TME and binds to adenosine receptors on the surface of other tumor cells [207].

Adenosine receptors such as A2AR and A2BR are G-coupled protein receptors that are present on cancer cells and most immune cells [212]. A2AR activation triggers a cAMP signaling cascade that affects several downstream pathways. In cancer cells, A2AR activation supports cancer cell growth [213] by activating PI3K-Akt signaling to resist apoptosis and promote epithelial to mesenchymal transition (EMT) [207,214]. However, in Teff cells, activation of A2AR-cAMP triggers PKA signaling activation, which induces immunosuppression and blocks cell growth via CREB activation and inhibition of NFkB and mTORC1 [206,207,215,216]. A2AR activation on CD4^+^ T cells inhibits IL-2 production and blocks upregulation of CD2 and CD28 [217]. In CD8^+^ T cells, A2AR activation inhibited TCR-Notch signaling and reduced IFNγ and granzyme B production [218]. In NK cells, A2AR activation downregulates OXPHOS and glycolysis [219] and inhibits their maturation and cytotoxic function [220]. A2AR activation induces FoxP3 expression to increase the differentiation and anti-inflammatory function of Treg cells [206,221]. A2AR activation also expands Treg populations, increases CTLA-4 expression, and enhances Treg immunosuppression [222]. MDSCs express surface CD39 and CD73 to produce extracellular adenosine [223], and adenosine-A2AR signaling induces M2 TAM polarization and promotes the generation of MDSCs [210,224,225]. In another study, adenosine receptor A2B activation altered DC differentiation to form an alternative population that expressed markers for both DC and macrophages [226]. This DC population was pro-tumorigenic and expressed several markers of angiogenesis, immune suppression, and immune tolerance [226].

### 3.10. Succinate and Itaconate

Succinate is a TCA metabolite that accumulates extracellularly in many cancers due to mitochondrial metabolic dysfunction or succinate dehydrogenase (SDH) mutations [227,228]. This excess succinate is secreted from cancer cells via monocarboxylate transporter 1 (MCT1) [229] into the TME and activates various signaling pathways in neighboring cells, either through accumulation within the cell or by activation of the succinate receptor GPR91 [228].

Succinate accumulation acts on cell signaling in several ways: First, by directly inhibiting prolyl-4-hydroxylase (PHD) and stabilizing HIF-1α expression [40]. Second, high intracellular succinate also acts as an epigenetic modifier in cells by disrupting the nuclear αKG/succinate ratio [230], which inhibits DNA demethylases such as the ten-eleven translocase (TET) family [75,231]. Third, succinate also inhibits TIL anti-tumor activity: Succinate is taken up by MCT1 in CD4^+^ and CD8^+^ T cells, and this accumulated succinate blocks metabolic flux through the TCA cycle by inhibiting the enzyme succinyl CoA synthetase (SUCLA2) [232]. These T cells showed reduced INFγ and TNFα production and degranulation in vitro [232].

Succinate receptor 1 (SUCNR1) is present on the surface of many tumor cells under hypoxia [233] and its activation triggers extracellular succinate uptake in target cells [234]. High succinate concentrations in the TME also increase the expression of SUCNR1 on many tumor cells [227], but CD4^+^ and CD8^+^ T cells instead downregulate SUCNR1 in response to succinate exposure [232], probably owing to its deleterious effects in these cells. The SUCNR1 receptor activates several downstream signaling cascades depending on cell type. Activation of SUCNR1 on TAMs, for instance, activates PI3K signaling and HIF-1α activation, triggering M2 TAM polarization and leading to immune suppression and cancer and TAM cell migration [227,228]. Stromal cell activation of SUCNR1 upregulates VEGF production through STAT3 and Erk1/2 signaling to increase vascularization of the tumor [235]. Activation of SUCNR1 has been reported in many cancer types and can activate several downstream pathways including Erk1/2 [236], prostaglandin E2 (PGE_2_) [233], p38 MAPK [237], Akt [233], and AMPK [227] with various effects. Ultimately, succinate promotes tumor growth by increasing cancer cell migration, EMT, invasion and metastasis, angiogenesis [233], and by inhibiting an effective anti-tumor immune response by inhibiting their cytotoxic and pro-inflammatory functions [227,228,232].

Itaconate is an interesting example of a metabolite that is produced almost exclusively by TAMs upon metabolic reprogramming [238]. Cis-aconitate is diverted from the TCA cycle and converted into itaconate by the enzyme cis-aconitate decarboxylase (IRG1), whose expression is induced in TAMs by pro-inflammatory cytokines [239]. Itaconate inhibits SDH, which blocks activity of the electron transport chain (ETC) complex II and reduces the rate of OXPHOS [240]. Itaconate’s inhibition of SDH also results in an accumulation of succinate, which in turn stabilizes HIF-1a expression by inhibiting PHD and promotes further metabolic reprogramming [241].

### 3.11. Exosomes

Cancers frequently upregulate secretion of tumor-derived exosomes (TDEs) as a result of low pH [242] and hypoxia-induced HIF-1α-dependent activation of Rab27a, a master regulator of exosome production [243]. TDEs secreted from cancer cells mimic the properties of their parent cell and can have activating or inhibitory effects on neighboring immune cells [244]. Tumors produce TDEs for the purpose of signaling to and conditioning other cells in the tumor to support tumorigenesis, growth, metastasis, and immunosuppression [245,246].

TDEs can facilitate communication between tumor cells in part through metabolic signaling. This can occur through several mechanisms including delivering enzymes to trigger a metabolic change in a target cell. In one example involving adenosine signaling, exosomes isolated from cancer cell lines and patient samples were found to express CD73 and CD39, and these TDE-associated phosphatases were responsible for as much as 20% of the ATP hydrolysis in the extracellular space, which resulted in inhibition of T cell proliferation and inflammatory cytokine production [247,248]. In another case, it was shown that TDEs from 5-fluorouracil (5FU)-resistant colorectal cancer (CRC) were enriched in the enzyme isocitrate dehydrogenase 1 (IDH1), which catalyzes the reversible reaction of converting isocitrate and NADP+ to αKG and NADPH [249,250]. These TDEs transmitted the IDH1 protein to non-resistant CRC cells, conferring 5FU resistance to the naïve cells by increasing their NADPH levels [250]. To date, several metabolic enzymes have been found in TDE [251] including enzymes involved in glycolysis (GLUT1 [252,253], HK2 [253], GAPDH [253], PGK1 [253], PKM2 [254,255,256]), PPP (G6PDH, TKT, TALDO1) [257], glutaminolysis (GLS1) [258], and the arginine metabolism enzyme Arg1 [259], to name just a few. Arg1 in particular is interesting because TDE-associated Arg1 was found to deplete the TME of arginine, which starved T cells and inhibited their anti-tumor effector functions [175].

Evidence also indicates TDEs shuttle metabolites between cancer cells, stromal cells, and immune cells within the tumor [260,261]. One study showed EVs from patient-derived CAFs from prostate and pancreatic tumors were enriched in the glycolytic metabolites lactate, pyruvate, and acetate; TCA intermediates like citrate, αKG, fumarate, and malate; several important amino acids including arginine, glutamate, glutamine, phenylalanine, and serine; and the lipid species stearate and palmitate [262]. Further, they were able to trace the redistribution of ^13^C-labeled metabolites from CAFs to cancer cells through CAF-derived extracellular vesicles (EVs) in vitro, and these metabolite-laden EVs inhibited OXPHOS and increased glycolysis and glutaminolysis in the targeted cancer cells [262]. In another study, TDEs were shown to contain adenosine, which diffused out of the vesicle in the extracellular space and inhibited perforin release from CD8^+^ T cells [263], thereby inhibiting their cytotoxic function.

The lipid composition of the TDE membrane also can trigger signaling responses in target cells. In one study, tumor-infiltrating DCs took up TDE from cervical cancer cells that were laden with long-chain fatty acids and stored those lipids as cytosolic lipid droplets [264]. This led to activation of PPARα, a master regulator of lipid metabolism, and activation of fatty acid oxidation (FAO) in the DCs, leading to inhibition of antigen presentation, failure to activate CD8^+^ T cells, and induction of Treg cells [264]. Several studies have shown the enrichment of various metabolites in exosomes from different cancer types, but much remains to be discovered about how TDE-delivered metabolites impact cells in the tumor.

### 3.12. Lipids

Lipid metabolism is one of the most frequently altered pathways in cancer [265]. Lipids serve several roles including in membrane synthesis, energy storage, and for producing signaling molecules [265,266]. Lipids aberrantly accumulate within tumor cells and cause disruption of anti-tumor functions. For example, cholesterol within the TME can accumulate in T cells, causing ER stress and blocking the synthesis and secretion of effector cytokines [267]. This ER stress activates the unfolded protein response (UPR) pathway XBP-1, which also promotes the expression of immunosuppressive molecules like PD-1, TIM-3, and LAG-3, which hampers the anti-tumor immune response and promotes T cell exhaustion [267]. Lipid accumulation is also associated with T cell dysfunction and exhaustion [267,268]. Because lipid accumulation triggers FAO and OXPHOS, lipids also have the effect of inducing a stronger memory phenotype and greater reactivation [269] of memory T cells (Tmem) and generate more Treg cells [270] than tumors that contain fewer lipids.

Similarly, many cancers exhibit aberrant lipid accumulation in tumor DCs [271] thanks in part to excess uptake of exogenous lipids via scavenger receptor A (CD204) [272], excess lipid synthesis by acetyl-CoA carboxylase-1 (ACC-1) [271], and through extracellular vesicle shuttling of lipids from cancer cells to DCs [264]. Lipid accumulation in tumor DCs results in an increase in FAO, which hinders an effective anti-tumor immune response by blocking antigen cross-presentation, blocking CD8^+^ T cell activation, and inducing Treg proliferation [264]. This lipid accumulation also triggers activation of PPARα, which drives excess lipid droplet biogenesis, FAO, and shifts the metabolism of the DC to OXPHOS, which causes immune dysfunction [264].

Enhanced lipid accumulation and metabolism also drive M2 TAM differentiation and activation by signaling through mitochondrial ROS-induced JAK1-STAT6 signaling [273]. In lymphoma, it was shown that excess lipids in the environment induced mitochondrial dysfunction in NK cells, suppressed cellular metabolism, and diminished IFNγ production and anti-tumor effector response [274]. MDSCs have also been observed to prefer FAO over glycolysis and upregulate FA uptake and storage compared to peripheral MDSCs [275]. Polyunsaturated fatty acids have also been shown to increase MDSC accumulation and immunosuppressive effects by activating JAK-STAT3 signaling [276]. CSCs also require both lipid synthesis and oxidation to maintain mitochondrial metabolism [158,277], and inhibition of fatty acid synthesis and oxidation both have been shown to reduce CSC numbers [277,278].

### 3.13. Methylglyoxal

MDSCs generate methylglyoxal from the spontaneous degradation of glycolytic glyceraldehyde-3-phosphate (G3P) and dihydroxyacetone phosphate (DHAP) [279,280]. Methylglyoxal accumulation suppresses glycolysis, which helps explain MDSCs’ low rate of metabolism compared to their progenitor cells [281]. Within the tumor, MDSCs perform cell-to-cell transfer of methylglyoxal to CD8^+^ T cells [282]. Within the T cell, methylglyoxal abrogates increased glucose uptake, glycolysis, and OXPHOS normally seen upon TCR activation [282]. Methylglyoxal also reacts with and depletes arginine from the cell, and because T cells require arginine for activation, this results in inhibition of TCR signaling and T cell activation and the abolition of TNFα, IFNγ, and granzyme B production [282].

## 4. Concluding Remarks

The role metabolic reprogramming plays in the progression of cancer is an area of intense study. It has been well established that many tumor cells, including cancer, immune, and stromal cells, adapt their metabolism to cope with limited nutrients and harsh TME conditions. Recently, the focus of cancer metabolism has expanded to include broader questions of how metabolism affects carcinogenesis and the anti-tumor immune response (Table 1). This has been reflected in the strategy of recent therapeutics, which have aimed to target specific metabolic enzymes and receptors with small molecules and monoclonal antibodies to disrupt metabolic signaling (Table 2). Alternatively, there have been some innovative approaches targeted at depleting essential nutrients from the TME by delivering active enzymes to the tumor using post-translational modifications or encapsulation within erythrocytes or live bacteria (Table 2). Given the efficacy of traditional small molecules and enzyme delivery technologies, the near future of TME metabolic research will likely expand on targeting metabolite production within and outside tumor cells as a direct therapy as well as to sensitize the tumor to immunotherapy. Ultimately, the goal of this review was to summarize some ways by which metabolites are used either as signaling molecules to affect other cells or by whose depletion the cancer cells inhibit the anti-tumor immune response (Table 1) in order to highlight how metabolism might be successfully targeted for current and future cancer therapies (Table 2). The result is a paracrine effect within the TME that targets critical signaling pathways in neighboring cells and favors cancer cell growth and attenuation of the immune response. The role metabolic signaling plays in the generation and progression of cancer warrants further attention.
Outstanding QuestionsGiven the established effects that several metabolites have on cell signaling pathways, such as lactate, glutamine, and adenosine, how do other TME metabolites impact pro-tumor signaling pathways and immune cell activation?How might the metabolic plasticity of the cancer cell help overcome potential nutrient deprivation therapies? Conversely, how will targeting conditionally essential pathways to the cancer cell affect other tumor cell types?

## Figures and Tables

**Table 1:** Summary of metabolites as signaling molecules.

Metabolite	Cancer Cell	Teff and Tmem Cells	NK Cell	Treg	TAM	DC	CAF	MDSC
Adenosine	Express surface CD39 and CD73 to produce extracellular adenosine from adenine nucleotides [129,130]. A2AR activation supports cell growth [133].	A2AR activation on CD4^+^ T cell inhibits IL-2 production, blocks upregulation of CD2 and CD28 [137]. A2AR activation inhibits TCR-Notch signaling, reduced IFNγ and Granzyme B production in CD8^+^ T cells [138].	A2AR activation inhibits maturation of NK cell [140]. A2AR activation downregulates OXPHOS, glycolysis [139].	Express surface CD39 and CD73 to produce extracellular adenosine from adenine nucleotides [129,130]. A2AR activation expands Treg population and increases CTLA-4 expression [142].	A2AR activation induces M2 TAM polarization [145].	A2AR activation inhibits antigen presentation [146].	A2AR activation supports cell growth [133].	MDSCs express surface CD39 and CD73 to produce extracellular adenosine from adenine nucleotides [143]. A2AR signaling promotes the generation of MDSCs [144].
Alanine	PDAC cells take up alanine from stellate cells and convert it to pyruvate to utilize it for TCA cycle [123]. Alanine-derived aKG is required for collagen hydroxylation in pre-metastatic niche tissue in breast cancer [124].	Extracellular alanine required for T cell activation. T cells upregulate alanine import rather than depending on synthesis from pyruvate. Alanine is directed towards protein synthesis. Alanine deprivation causes failure to leave quiescence [125]. Reactivation of Tmem cells requires extracellular alanine [125].	- - -	- - -	- - -	- - -	PDAC stellate cells upregulate autophagy to secrete alanine to cancer cells [123].	- - -
Arginine and Polyamines	ASS1 deficiency causes dependence on extracellular arginine, causing depletion from the TME [87]. Take up exogenous arginine for polyamine synthesis and nitric oxide production [88].	Depletion of arginine from TME impairs Teff function [91,96]. Arginine depletion from TME inhibits Teff mTORC1 activity, decreases effector functions and promotes memory phenotype [94,100].	Arginine depletion inhibits cytotoxicity, IFN-g production, and viability of NK cells [101].	IDO expression depletes arginine from TME, which inhibits Teff and promotes Treg [96].	M2 TAMs increase expression and secretion of Arg1 and deplete arginine from the TME to inhibit effector T cells [97]. TGFb signaling and HIF-1a-induced metabolic reprogramming upregulate arginine metabolism [45,93]. M2 TAMs secrete polyamines to promote cell division in cancer cells [45].	IDO expression depletes arginine from TME, which inhibits Teff and promotes Treg [96].	TGFb signaling and HIF-1a-induced metabolic reprogramming upregulate arginine metabolism [45,93]. IDO expression depletes arginine from TME, which inhibits Teff and promotes Treg [96].	Secrete Arg1 into TME to deplete arginine and inhibit T cell anti-tumor function [99]. Arginine depletion upregulates expression of VISTA, CD39L1 immunosuppressive molecules [102].
Cysteine	Cancer cells upregulate cystine/glutamate antiporter xCT to resist ferroptosis, which depletes cysteine from the TME as a result [118]. Deplete cysteine from TME, which limits TIL activation and effector function [117].	Naïve T cells rely on cysteine secreted from macrophages and DCs and take up cysteine via the neutral amino acid transporter ASC rather than importing cystine via xCT [119]. TIL activation and effector function is hampered by cysteine depletion by cancer cells and MDSCs [117].	- - -	Inhibit extracellular DC-mediated cysteine production from GSH in order to inhibit Teff function [120].	Secrete cysteine for utilization by naïve T cells [119].	Supply cysteine for utilization by naïve T cells by secreting GSH, which is cleaved into cysteine in the extracellular space [119,120].	Stromal cells upregulate xCT to produce excess GSH, which is transferred to cancer cells and promotes chemoresistance [202].	Deplete cysteine from TME, which limits TIL activation and effector function [117].
Exosomes	Upregulate tumor-derived exosome (TDE) production in response to low pH and activation of HIF-1a/Rab27a [163,164]. TDE-associated CD39 and CD73 account for as much as 20% of extracellular ATP hydrolysis and adenosine production [168]. TDE from 5-fluorouracil (5FU)-resistant cancer cells transmitted isocitrate dehydrogenase (IDH1) to non-resistant cells, which induced 5FU resistance through increased NADPH production [171]. Several metabolic enzymes have been identified in TDEs including glycolytic, pentose phosphate pathway, and glutaminolytic enzymes [106,173,174,175,176,177,178,179,180]. Melanoma TDE sensitize pre-metastatic niche sites in lung epithelia by inhibiting the enzyme CH25H from producing 25-hydroxycholesterol (25HC) and inducing degradation of IFNg receptor IFNAR1 [203].	Tumor-derived exosomes (TDEs) express Fas ligand and deliver to T cells to induce apoptosis [204]. TDE express TGFb, which inhibits CD25 (IL-2 receptor)-induced cell growth and cytolytic function in T cells and suppresses their activation by downregulating the expression of the TGFb receptor NKG2D [168,205]. TDE-associated adenosine inhibits T cell proliferation, inflammatory cytokine production, and perforin release [169,184]. TDE-associated arginase 1 (Arg1) depleted arginine from the TME, starving T cells and inhibiting their anti-tumor functions [94].	Tumor-derived exosomes (TDEs) express TGFb, which suppresses NK cell activation by downregulating the expression of the TGFb receptor NKG2D [168].	Tumor-derived exosomes (TDE) express TGFb, which activates and expands Treg cells [168].	TAM-derived exosomes deliver lncRNA HISLA to cancer cells, which stabilizes HIF-1a expression, upregulates glycolysis and lactate production, and promotes chemoresistance in cancer cells [206]. TDEs condition tissue resident macrophages in pre-metastatic site by activating TLR2-NFkB signaling, which upregulates Arg1 and VEGF, increases NOS expression, inhibits OXPHOS, and increases glycolysis and lactate production [207].	Tumor-infiltrating DCs take up long chain fatty acids from TDEs, which activated PPARa and FAO leading to inhibition of DC antigen presentation, failure to activate CD8^+^ T cells, and induction of Treg cells [185].	Tumor-derived exosomes (TDEs) express TGFb, which transforms normal fibroblasts to cancer-associated fibroblasts (CAFs) [208]. TDE deliver miR-105 to reprogram CAF glucose and glutamine metabolism towards a catabolic phenotype to feed adjacent cancer cells and to detoxify TME waste [209]. CAF-derived exosomes are enriched in several glycolytic intermediates, TCA metabolites, amino acids, and lipids, and deliver these metabolites to cancer cells where they inhibit OXPHOS and increase glycolysis and glutaminolysis [183].	- - -
Glucose	Third highest consumer of glucose in the tumor [4]. Perform aerobic glycolysis where glucose metabolism is decoupled from mitochondrial metabolism and is instead converted to lactate [2]. Glucose is often depleted from poorly vascularized regions of the tumor [7]. High glycolytic flux promotes G-CSF secretion and recruitment of MDSCs [12].	Second highest consumer of glucose in the tumor [4]. Perform aerobic glycolysis where glucose metabolism is decoupled from mitochondrial metabolism and is instead converted to lactate [3]. Glucose limitation blocks effector cytokine production [9].	Uneducated NK cells mainly use OXPHOS for energy production. Educated NK cells upregulate glycolysis in addition to OXPHOS for energy production and cytotoxic functions [210].	Low glucose induces FoxP3 expression, which converts anti-tumor CD4^+^ Teff cells into pro-tumor Treg cells [10]. Treg growth and suppressive function is inhibited by high glucose [11].	M1 TAMs upregulate aerobic glycolysis in response to the harsh TME environment [211]. M2 TAMs further upregulate glycolysis and lactate production [159].	Activated DCs upregulate glycolysis and lactate production [55].	Hyperglycemia activates PDAC stellate cell CXCL12 production, binds CXCR4 receptor on cancer cell to activate MAPK and induces proliferation and migration [13].	Highest consumer of glucose in the tumor [4].
Glutamine	Highest consumer of glutamine in the tumor [4]. Glutamine becomes conditionally essential to many cancers [60]. Glutaminolysis regulates mTOR signaling, redox balance, autophagy, apoptosis and ferroptosis [64,65,66,67].	Teff cells increase glutaminolysis to support the TCA cycle and biosynthesis of cellular components [212].	Require sustained glutaminolysis to maintain Myc expression [75].	Treg differentiation by FoxP3 is inhibited by glutaminolysis [74].	M1 TAMs have a truncated TCA cycle due to inactivation of IDH and SDH, and require glutamine as an alternative carbon source [213]. M1 TAMs require glutaminolysis for cytokine production, antigen presentation, and phagocytosis [72]. M2 TAMs upregulate glutaminolysis to produce excess alpha-ketoglutarate to reinforce M2 polarization through epigenetic reprogramming [73].	Glutaminolysis maintains redox metabolism and supports DC activation and antigen presentation [78].	CAFs produce and secrete excess glutamine from branched chain amino acids and aspartate for use by cancer cells [70].	Increased glutamine consumption in tumor cells increases expression of LAP and CSF3, which promotes recruitment and generation of MDSCs [71].
Lactate	Many cancer types consume lactate as fuel when glucose is low [20,21]. Exogenous lactate acts in a paracrine fashion on neighboring cancer cells to stabilize HIF-1a/2a, activate c-Myc, and upregulate glutaminolysis [28,29].	CD8^+^ T cells can consume lactate as fuel when glucose is low [23]. High extracellular lactate disrupts T cell glycolysis and mitochondrial metabolism and causes a net influx of lactate that lowers intracellular pH [33,34,35]. High extracellular lactate induces apoptosis in T and NK cells, limits effector functions and inhibits NFAT-mediated expression of proinflammatory cytokines [35,38]. High lactate disrupts chemotaxis and migration of CD4^+^ and CD8^+^ T cells and limits CD8^+^ release of perforin and granzyme [39].	High extracellular lactate induces apoptosis in T and NK cells, limits effector functions and inhibits NFAT-mediated expression of proinflammatory cytokines [35,38].	High lactate in the TME induces a switch from glycolysis to OXPHOS, which maintains Treg immunosuppressive function by allowing them to consume lactate [11]. The Treg transcription factor FoxP3 supports growth under low glucose high lactate environments by transcriptionally repressing Myc, suppressing glycolysis, increasing the rate of OXPHOS, and reversing the reaction catalyzed by LDH to consume lactate and recycle and maintain a pool of NAD+ [40]. Lactate induces Treg PD-1 expression through the transcription factor NFAT [43].	High lactate in TME drives M2 TAM polarization by stabilizing HIF-1a expression, which induces M2 gene expression and polarization and promotes a switch from glycolytic metabolism to OXPHOS via lactic acid and lipid oxidation [45]. Lactate-HIF-1a signaling in TAMs drives significant VEGF expression and promotes vascularization of the tumor [45]. Lactylation modification of histones converts TAMs from the M1 to M2 phenotype [46]. Lactate inhibits expression of ATP6V0d2, preventing the degradation of HIF-2a in lysosomes and maintaining M2 HIF functions [47]. Lactate increases PD-L1 expression on TAM [48].	High extracellular lactate prevents the diffusion of glycolytic pyruvate, and this accumulated lactate shifts the DC into a tolerogenic phenotype upon TLR stimulation [57].	CAF respond to tumor-derived proinflammatory cytokines by producing and secreting lactate into the TME [49]. CAF upregulation of glycolytic lactate secretion can occur through hypoxia-induced expression of glycolytic genes via HIF-1a [49]. Cell-cell contacts with cancer cells activates SIRT3 ROS signaling in CAFs that upregulates MCT4 lactate export [49,50,51].	Lactate receptor GPR81 activates MDSCs and generates resistance to radiotherapy by signaling through mTOR, HIF-1a and STAT3 [59].
Lipids	The signaling lipid prostaglandin E2 (PGE2) is the most abundant prostaglandin found in tumors [187]. PGE2 is produced by the enzyme COX2 in cancer cells and stromal cells and promotes inflammation and tumor growth by signaling through several signaling pathways (Ras, Erk, GSK3b, b-Catenin, and PPARd) in an autocrine and paracrine fashion [214,215,216,217].	Lipid accumulation triggers FAO and OXPHOS, which induces a stronger memory phenotype and greater reactivation of T cells [190]. Cholesterol originating in the TME can accumulate in T cells, causing ER stress and blocking the synthesis and secretion of effector cytokines. This ER stress activates the unfolded protein response (UPR) pathway XBP-1, which also promotes the expression of immunosuppressive molecules like PD-1, TIM-3, and LAG-3, which hampers the anti-tumor immune response and promotes T cell dysfunction and exhaustion [188,189].	Excess lipid metabolism induced NK cell mitochondrial dysfunction, suppressed cellular metabolism, and diminished IFN-g production and anti-tumor effector response [195].	Lipid accumulation triggers FAO and OXPHOS, which generates more Treg cells than in tumors that contain fewer lipids [191].	Enhanced lipid accumulation and metabolism drive M2 TAM differentiation and activation through mitochondrial ROS-induced JAK1-STAT6 signaling [194].	Upregulation of CD204 in DC causes an increase in uptake of extracellular lipids, which hampers antigen processing and presentation [192]. TDE deliver lipids from cancer cells to DC, causing lipid accumulation, activation of PPARa, excess lipid droplet biogenesis, FAO, and shifts the metabolism of the DC to OXPHOS, which causes immune dysfunction [185].	PGE2 is produced by the enzyme COX2 in cancer cells and stromal cells and promotes inflammation and tumor growth by signaling through one of several signaling pathways in an autocrine and paracrine fashion [218,219].	Tumor MDSCs show increased FA uptake, storage, and oxidation compared to peripheral MDSCs [196]. PUFA activate JAK-STAT3 signaling to drive MDSC accumulation and immunoinhibitory function [197].
Methionine	Many cancers are addicted to methionine, and cancer cells maximize their methionine uptake by upregulating the methionine transporter SLC43A2 in addition to increasing production from their endogenous methionine cycle [103,104,105].	Methionine addiction by cancer cells limits its availability for T cells in the tumor, resulting in low S-adenosylmethionine (SAM) production and the loss of dimethylation at histone H3K79me2. This alters epigenetic control and in turn blocks T cell immunity by reducing expression of STAT5-derived cytokines [105].	- - -	- - -	- - -	- - -	- - -	- - -
Methylglyoxal	- - -	Methylglyoxal acquired from MDSCs suppresses the metabolic changes that normally occur upon TCR activation including increased glucose uptake, glycolysis, and OXPHOS [201]. Methylglyoxal reacts with and depletes arginine from the T cell, which inhibits TCR signaling and T cell activation and abolishes TNFa, IFNg, and granzyme B production [201].	- - -	- - -	- - -	- - -	- - -	MDSCs generate methylglyoxal as a by-product of glucose metabolism, and its accumulation suppresses glycolysis in MDSCs [198,199,200]. MDSCs directly contact and perform cell-to-cell transfer of methylglyoxal into CD8^+^ T cells [201].
Succinate	Succinate accumulates extracellularly in many cancers due to mitochondrial metabolic dysfunction, succinate dehydrogenase (SDH) mutations, and secretion from cancer cells by MCT-1 [147,148,149]. Succinate promotes cancer cell migration, EMT, invasion and metastasis, and angiogenesis [154]. Succinate activates the succinate receptors GPR91 and SUCNR1 on tumor cells [148]. SUCNR1 activation triggers extracellular succinate uptake and activates several downstream signaling pathways including Erk1/2, prostaglandin E2 (PGE2), p38 MAPK, Akt, and AMPK with various effects [147,154,155,157,158]. Succinate accumulation directly inhibits PHD and stabilizes HIF-1a expression [150]. High intracellular succinate disrupts the nuclear aKG/succinate ratio, which inhibits TET family DNA demethylases [97,151,152].	Succinate is taken up by MCT-1 in CD4^+^ and CD^8^+ T cells, and this accumulated succinate blocks metabolic flux through the TCA cycle by inhibiting the enzyme succinyl CoA synthetase (SUCLA2) [153]. High succinate inhibits TIL anti-tumor activity by reducing INFg and TNFa production and degranulation [153]. CD4^+^ and CD8^+^ T cells downregulate SUCNR1 in response to succinate exposure [153].	- - -	- - -	Activation of SUCNR1 on TAMs activates PI3K signaling and HIF-1a activation, triggering M2 TAM polarization and leading to immune suppression and cancer and TAM cell migration [147,148].	- - -	Stromal cell activation of SUCNR1 upregulates VEGF production through STAT3 and Erk1/2 signaling to increase vascularization of the tumor [156].	- - -
Tryptophan	Exogenous tryptophan is required to maintain kynurenine pathway, NAD+ synthesis [79]. Express IDO to deplete extracellular tryptophan and inhibit T cell proliferation [81].	Tryptophan depletion in the TME starves TILs, which induces GCN2 activation and mTOR inhibition and leads to anergy and cell cycle arrest [81]. Kynurenine produced from tryptophan activates the AhR receptor in CD4^+^ T cells, which causes them to differentiate into Tregs [81,82]. Kynurenine induces PD-1 expression on CD8^+^ T cells [83].	- - -	Express IDO to deplete extracellular tryptophan, which inhibits Teff function and promotes Tregs [96].	- - -	Express IDO to deplete extracellular tryptophan, which inhibits Teff function and promotes Tregs [96].	Express IDO to deplete extracellular tryptophan, which inhibits Teff function and promotes Tregs [96].	- - -

**Table 2:** Summary of clinical trials evaluating metabolism-targeting agents in cancer.

Metabolic Pathway	Trial ID	Drug/Treatment	Target and Impact on TME	Cancer Type	Phase	Status
GLUCOSE	NCT05957939	Alkaline glucosodienes	Block glycolysis, raise pH	Triple-negative breast cancer	Phase I	Not yet recruiting
	NCT01935531	Diclofenac	Inhibit MYC, glycolysis, and lactate transport; lower lactate levels	Actinic keratosis	Phase I	Completed
	NCT04114136	Metformin, Rosiglitazone + anti-PD-1/PD-L1 mAB	Block glucose metabolism, sensitize to anti-PD-1 mAB	Multiple solid tumors	Phase II	Recruiting
	NCT04542291	Dapagliflozin	Sodium-glucose cotransporter-2 (SGLT2)	Pancreatic cancer	Phase I	Completed
	NCT01205672	Metformin	Glucose metabolism, mTOR	Endometrial cancer	Phase I	Completed
	NCT03763396	Ketoconazole, Posaconazole	Hexokinase 2, glucose metabolism	Glioma	Phase I	Not yet recruiting
	NCT01620593	Metformin, castration	Inhibit glucose metabolism	Prostate cancer	Phase II	Completed
GLUTAMINE	NCT02071888	Telaglenastat	Glutaminase inhibitor	Hematological Tumors	Phase I	Completed
	NCT02071862	Telaglenastat	Glutaminase inhibitor	Multiple solid tumors	Phase I	Completed
	NCT02071927	Telaglenastat	Glutaminase inhibitor	Leukemia	Phase I	Completed
	NCT03872427	Telaglenastat	Glutaminase inhibitor	Multiple solid tumors	Phase II	Active, not recruiting
	NCT04250545	Telaglenastat plus Sapanisertib	Glutaminase, mTOR	Squamous cell lung cancer, non-small cell lung cancer	Phase I	Active, not recruiting
	NCT03528642	Telaglenastat plus radiation and Temozolomide	Glutaminase, DNA replication	IDH-Mutated Diffuse Astrocytoma or Anaplastic Astrocytoma	Phase I	Active, not recruiting
	NCT03831932	Telaglenastat and Osimertinib	Glutaminase, EGFR	EGFR-mutated stage IV non-small cell lung cancer	Phase I/II	Active, not recruiting
	NCT03428217	Telaglenastat plus Cabozantinib	Glutaminase, VEGF	Renal cell carcinoma	Phase II	Completed
	NCT03057600	Telaglenastat plus Paclitaxel	Glutaminase, mitosis	Triple-negative breast cancer	Phase II	Completed
	NCT03163667	Telaglenastat plus Everolimus	Glutaminase, mTOR	Renal cell carcinoma	Phase II	Completed
	NCT04471415	DRP-104	Glutamine metabolism	Non-small cell lung cancer	Phase I/II	Terminated
	NCT06027086	DRP-104 plus Durvalumab	Glutamine metabolism, enhance anti-PD-L1 therapy	Fibrolamellar Carcinoma	Phase Ib/II	Recruiting
ASPARAGINE	NCT01523808	L-Asparaginase-erythrocyte suspension	Asparagine metabolism	Pancreatic adenocarcinoma	Phase I	Completed
	NCT03674242	Eryaspase (L-Asparaginase-erythrocyte suspension) plus Gemcitabine and Carboplatin	Asparagine metabolism, DNA replication	Triple-negative breast cancer	Phase II/III	Terminated
	NCT01523782	GRASPA (L-Asparaginase-erythrocyte suspension)	Asparagine metabolism	Acute Lymphoblastic Leukemia	Phase II	Completed
	NCT01810705	GRASPA (L-Asparaginase-erythrocyte suspension) plus cytarabine	Asparagine metabolism, DNA replication	Acute Lymphoblastic Leukemia	Phase II	Completed
	NCT01251809	Oncaspar and Pegaspargase (pegylated recombinant asparaginase)	Asparagine metabolism	Acute Lymphoblastic Leukemia	Phase I/II	Terminated
	NCT04953780	Calaspargase pegol-mknl (pegylated recombinant asparaginase) plus cytarabine and idarubicin	Asparagine metabolism, DNA replication	Acute Myeloid Leukemia	Phase I	Active, not recruiting
ARGININE	NCT05759923	OATD-02	Arginase 1/2 inhibitor	Multiple solid tumors	Phase I	Recruiting
	NCT03236935	NG-monomethyl-L-arginine (L-NMMA) and pembrolizumab	Nitric oxide synthase inhibitor, anti-PD-1	Multiple solid tumors	Phase I	Active, not recruiting
	NCT02285101	PEG-BCT-100 (pegylated arginase)	Arginine metabolism	Melanoma	Phase I	Completed
	NCT00988195	PEG-BCT-100 (pegylated arginase)	Arginine metabolism	Hepatocellular carcinoma	Phase I	Completed
	NCT03455140	PEG-BCT-100 (pegylated arginase)	Arginine metabolism	Multiple solid and liquid tumors	Phase I/II	Completed
	NCT01092091	PEG-BCT-100 (pegylated arginase)	Arginine metabolism	Hepatocellular carcinoma	Phase II	Completed
	NCT00029900	ADI-PEG (pegylated arginine deiminase)	Arginine metabolism	Metastatic melanoma	Phase I	Completed
	NCT01266018	ADI-PEG 20 (pegylated arginine deiminase)	Arginine metabolism	Small cell lung cancer	Phase II	Terminated
	NCT01497925	ADI-PEG 20 (pegylated arginine deiminase) plus Docetaxel	Arginine metabolism, DNA replication	Prostate cancer, non-small cell lung cancer	Phase I	Completed
	NCT02102022	ADI-PEG 20 (pegylated arginine deiminase) plus FOLFOX	Arginine metabolism, DNA replication	Hepatocellular carcinoma	Phase I/II	Terminated
	NCT01665183	ADI-PEG 20 (pegylated arginine deiminase) plus Cisplatin	Arginine metabolism, DNA replication	Metastatic melanoma	Phase I	Completed
	NCT03254732	ADI-PEG 20 (pegylated arginine deiminase) plus Pembrolizumab	Arginine metabolism, anti-PD-1	Multiple solid tumors	Phase Ib	Terminated
	NCT01948843	ADI-PEG 20 (pegylated arginine deiminase) plus Doxorubicin	Arginine metabolism, DNA repair	HER2 negative metastatic breast cancer	Phase I	Completed
	NCT06085729	ADI-PEG 20 (pegylated arginine deiminase) plus Carboplatin and Cabazitaxel	Arginine metabolism, DNA replication	Prostate cancer	Phase I/II	Recruiting
	NCT02029690	ADI-PEG 20 (pegylated arginine deiminase) plus Pemetrexed and Cisplatin	Arginine metabolism, folate metabolism, DNA replication	Multiple solid tumors	Phase I	Terminated
	NCT03922880	ADI-PEG 20 (pegylated arginine deiminase) plus Nivolumab and Ipilimumab	Arginine metabolism, anti-PD-1, anti-CTLA-4	Uveal melanoma	Phase I	Completed
	NCT02101593	ADI-PEG 20 (pegylated arginine deiminase) plus Sorafenib	Arginine metabolism, cell signaling	Hepatocellular carcinoma	Phase I	Completed
	NCT05001828	ADI-PEG 20 (pegylated arginine deiminase) plus Venetoclax and Azacitidine	Arginine metabolism, BCL-2 inhibition, DNA replication	Acute myeloid leukemia	Phase I	Recruiting
	NCT02101580	ADI-PEG 20 (pegylated arginine deiminase) plus Nab-Paclitaxel and Gemcitabine	Arginine metabolism, DNA replication	Pancreatic cancer	Phase Ib	Completed
	NCT05616624	ADI-PEG 20 (pegylated arginine deiminase) plus Gemcitabine and Docetaxel	Arginine metabolism, DNA replication	Small cell lung cancer, non-small cell lung cancer	Phase I/II	Recruiting
	NCT00520299	ADI-PEG 20 (pegylated arginine deiminase)	Arginine metabolism	Melanoma	Phase I/II	Completed
	NCT05813327	ADI-PEG 20 (pegylated arginine deiminase) plus Ifosfamide and radiotherapy	Arginine metabolism, DNA replication	Sarcoma	Phase I/II	Recruiting
	NCT03449901	ADI-PEG 20 (pegylated arginine deiminase) plus Gemcitabine and Docetaxel	Arginine metabolism, DNA replication	Sarcomas, small cell lung cancer	Phase II	Completed
	NCT00056992	ADI-PEG 20 (pegylated arginine deiminase)	Arginine metabolism	Hepatocellular carcinoma	Phase II	Completed
	NCT04587830	ADI-PEG 20 (pegylated arginine deiminase) plus Radiotherapy and Temozolomide	Arginine metabolism, DNA replication	Glioblastoma multiforme	Phase II	Recruiting
	NCT01910012	ADI-PEG 20 (pegylated arginine deiminase)	Arginine metabolism	Acute myeloid leukemia	Phase II	Completed
	NCT01910025	ADI-PEG 20 (pegylated arginine deiminase)	Arginine metabolism	Non-Hodgkin’s lymphoma	Phase II	Completed
	NCT00450372	ADI-PEG 20 (pegylated arginine deiminase)	Arginine metabolism	Metastatic melanoma	Phase II	Completed
	NCT02006030	ADI-PEG 20 (pegylated arginine deiminase) plus transarterial chemoembolization	Arginine metabolism	Hepatocellular carcinoma	Phase II	Completed
	NCT06034977	ADI-PEG 20 (pegylated arginine deiminase) plus Lenvatinib	Arginine metabolism, kinase inhibition	Hepatocellular carcinoma	Phase II	Recruiting
	NCT02709512	ADI-PEG 20 (pegylated arginine deiminase) plus Pemetrexed and Cisplatin	Arginine metabolism, folate metabolism, DNA replication	Malignant pleural mesothelioma	Phase II/III	Completed
	NCT05712694	ADI-PEG 20 (pegylated arginine deiminase) plus Gemcitabine and Docetaxel	Arginine metabolism, DNA	Leiomyosarcoma	Phase III	Recruiting
	NCT01287585	ADI-PEG 20 (pegylated arginine deiminase)	Arginine metabolism	Hepatocellular carcinoma	Phase III	Completed
	NCT05317819	ADI-PEG 20 (pegylated arginine deiminase)	Arginine metabolism	Hepatocellular carcinoma	Phase III	Recruiting
TRYPTOPHAN	NCT03364049	MK-7162 plus Pembrolizumab	Indoleamine 2, 3-dioxygenase (IDO) inhibitor, anti-PD-1	Multiple solid tumors	Phase I	Completed
	NCT03792750	BMS-986205 alone or in combination with Nivolumab	Indoleamine 2, 3-dioxygenase (IDO) inhibitor, anti-PD-1	Multiple solid tumors	Phase I/II	Completed
	NCT03516708	Epacadostat plus radiation, CAPOX, FOLFOX	Indoleamine 2, 3-dioxygenase (IDO) inhibitor	Rectal cancer	Phase I/II	Recruiting
	NCT00567931	Indoximod (1-methyl-D-tryptophan)	Indoleamine 2, 3-dioxygenase (IDO) inhibitor	Multiple solid tumors	Phase I	Completed
	NCT01191216	Indoximod (1-methyl-D-tryptophan) and Docetaxel	Indoleamine 2, 3-dioxygenase (IDO) inhibitor	Multiple solid tumors	Phase I	Completed
	NCT02502708	Indoximod (1-methyl-D-tryptophan) plus Temozolomide	Indoleamine 2, 3-dioxygenase (IDO) inhibitor, DNA repair	Malignant brain tumor	Phase I	Completed
	NCT02835729	Indoximod (1-methyl-D-tryptophan) plus cytarabine and idarubicin	Indoleamine 2, 3-dioxygenase (IDO) inhibitor, DNA replication	Acute myeloid leukemia	Phase I	Completed
	NCT01042535	Indoximod (1-methyl-D-tryptophan) and Ad.p53 DC vaccine	Indoleamine 2, 3-dioxygenase (IDO) inhibitor, immunotherapy	Metastatic breast cancer	Phase I/II	Completed
	NCT02052648	Indoximod (1-methyl-D-tryptophan) plus Temozolomide	Indoleamine 2, 3-dioxygenase (IDO) inhibitor, DNA repair	Malignant brain tumor	Phase I/II	Completed
	NCT02077881	Indoximod (1-methyl-D-tryptophan) plus Gemcitabine and Nab-Paclitaxel	Indoleamine 2, 3-dioxygenase (IDO) inhibitor, DNA replication	Metastatic pancreatic cancer	Phase I/II	Completed
	NCT02073123	Indoximod (1-methyl-D-tryptophan) plus immune checkpoint inhibitors	Indoleamine 2, 3-dioxygenase (IDO) inhibitor, immune checkpoint	Metastatic melanoma	Phase I/II	Completed
	NCT01560923	Indoximod (1-methyl-D-tryptophan) and Provenge	Indoleamine 2, 3-dioxygenase (IDO) inhibitor, immunotherapy	Castration-resistant prostate cancer	Phase II	Completed
	NCT01792050	Indoximod (1-methyl-D-tryptophan) plus docetaxel or paclitaxel	Indoleamine 2, 3-dioxygenase (IDO) inhibitor, DNA replication	Metastatic breast cancer	Phase II	Completed
ADENOSINE	NCT04381832	Etrumadenant	A2a, A2b receptor antagonist	Metastatic castrate-resistant prostate cancer	Phase I/II	Completed
	NCT05024097	Etrumadenant plus Zimberelimab and radiation	A2a, A2b receptor antagonist; anti-PD-1 antibody	Rectal cancer	Phase II	Recruiting
	NCT05915442	Quemliclustat plus Etrumadenant and Zimberelimab	CD73 antagonist; A2a, A2b receptor antagonist; anti-PD-1 antibody	Prostate cancer	Phase II	Recruiting
	NCT05886634	Etrumadenant and Zimberelimab	A2a, A2b receptor antagonist; anti-PD-1 antibody	Liposarcoma	Phase II	Recruiting
	NCT04660812	Etrumadenant plus several drug combinations	A2a, A2b receptor antagonist	Metastatic colorectal cancer	Phase I/II	Active, not recruiting
	NCT06048484	Quemliclustat with Etrumadenant and Zimberelimab and radiation	CD73 inhibitor; A2a, A2b receptor antagonist; anti-PD-1 antibody	Pancreatic ductal adenocarcinoma	Phase II	Recruiting
	NCT05688215	Quemliclustat and Zimberelimab	CD73 inhibitor; anti-PD-1 antibody	Pancreatic adenocarcinoma	Phase I/II	Recruiting
	NCT04306900	TTX-030	Anti-CD39 antibody	Multiple solid tumors	Phase I	Completed
	NCT03884556	TTX-030	Anti-CD39 antibody	Multiple solid tumors	Phase I	Completed
	NCT06119217	TTX-030 with nab-paclitaxel, gemcitabine, and budigalimab	Anti-CD39 antibody; mitotic arrest; DNA synthesis; anti-PD-1	Pancreatic adenocarcinoma	Phase II	Active, not recruiting
	NCT05272709	TT-702	A2b receptor antagonist	Multiple solid tumors	Phase I/II	Recruiting
	NCT04969315	TT-10	A2a, A2b receptor antagonist	Multiple solid tumors	Phase I/II	Active, not recruiting
	NCT02655822	Ciforadenant alone or with Atezolizumab	A2a receptor antagonist; anti-PD-L1 antibody	Multiple solid tumors	Phase I	Completed
	NCT05501054	Ciforadenant with Ipilimumab and Nivolumab	A2a receptor antagonist; anti-CTLA-4 antibody; anti-PD-1 antibody	Renal cell carcinoma	Phase I/II	Recruiting
	NCT05117177	Inupadenant	A2a receptor antagonist	Multiple solid tumors	Phase I	Completed
	NCT05403385	Inupadenant plus chemotherapy	A2a receptor antagonist	Non-small cell lung cancer	Phase II	Recruiting
	NCT02403193	Taminadenant alone or with PDR001	A2a receptor antagonist; anti-PD-1 antibody	Non-small cell lung cancer	Phase I	Completed
	NCT04089553	AZD4635 plus Durvalumab or Oleclumab	A2a receptor antagonist; anti-PD-L1 antibody; anti-CD73 antibody	Prostate cancer	Phase II	Completed
	NCT03274479	PBF-1129	A2b receptor antagonist	Non-small cell lung cancer	Phase I	Active, not recruiting
	NCT05234307	PBF-1129 and Nivolumab	A2b receptor antagonist; anti-PD-1 antibody	Non-small cell lung cancer	Phase I	Recruiting
	NCT04336098	SRF617	Anti-CD39 antibody	Multiple solid tumors	Phase I	Completed
	NCT03454451	Mupadolimab alone or with Ciforadenant and/or Pembrolizumab	Anti-CD73 antibody; A2a receptor antagonist; anti-PD-1 antibody	Multiple solid tumors	Phase I	Completed
	NCT03616886	Oleclumab with Paclitaxel, Carboplatin, and Durvalumab	Anti-CD73 antibody; mitotic arrest; DNA damage; anti-PD-L1	Triple-negative breast cancer	Phase I/II	Phase I/II
	NCT03773666	Oleclumab with Durvalumab	Anti-CD73 antibody; anti-PD-L1 antibody	Bladder cancer	Phase I	Completed
	NCT05270213	RBS2418	Ectonucleotide pyrophosphatase/phosphodiesterase I (ENPP1) inhibitor	Multiple solid tumors	Phase I	Recruiting
POLYAMINES	NCT05717153	Difluoromethylornithine (DFMO) and AMXT 1501	Ornithine decarboxylase inhibitor; polyamine transport inhibitor	Glioma	Phase I	Recruiting
	NCT03536728	Difluoromethylornithine (DFMO) and AMXT 1501	Ornithine decarboxylase inhibitor; polyamine transport inhibitor	Multiple solid tumors	Phase I	Completed
	NCT02030964	Difluoromethylornithine (DFMO) with Celecoxib, Cyclophosphamide, and Topotecan	Ornithine decarboxylase inhibitor; chemotherapeutics	Neuroblastoma	Phase I	Active, not recruiting
	NCT05500508	Difluoromethylornithine (DFMO) and AMXT 1501	Ornithine decarboxylase inhibitor; polyamine transport inhibitor	Multiple solid tumors	Phase I/II	Active, not recruiting
	NCT06059118	Difluoromethylornithine (DFMO) with Testosterone and Enzalutamide	Ornithine decarboxylase inhibitor; hormone; anti-androgen	Prostate cancer	Phase II	Recruiting
	NCT00293488	SL-11047	Polyamine analog	Lymphoma	Phase I	Completed
	NCT00086736	Eflornithine and Bicalutamide	Ornithine decarboxylase inhibitor; anti-androgen	Prostate cancer	Phase II	Completed
	NCT03794349	Eflornithine and Irinotecan Temozolomide, and Dinutuximab	Ornithine decarboxylase inhibitor; chemotherapeutics	Neuroblastoma	Phase II	Active, not recruiting
	NCT05254171	SBP-101 with Nab-Paclitaxel and Gemcitabine	Polyamine analog; chemotherapeutics	Pancreatic cancer	Phase II/III	Recruiting
METHIONINE	NCT06568614	SYH2039	Methionine adenosyltransferase 2 alpha (MAT2A) inhibitor	Multiple solid tumors	Phase I	Not yet recruiting
	NCT04794699	IDE397	Methionine adenosyltransferase 2 alpha (MAT2A) inhibitor	Multiple solid tumors	Phase I	Recruiting
	NCT06414460	ISM3412	Methionine adenosyltransferase 2 alpha (MAT2A) inhibitor	Multiple solid tumors	Phase I	Not yet recruiting
	NCT05038150	SGN1 (engineered *Salmonella* bacteria overexpressing L-methioninase)	Methionine metabolism	Multiple solid tumors	Phase I/II	Recruiting
	NCT05103345	SGN1 (engineered *Salmonella* bacteria overexpressing L-methioninase)	Methionine metabolism	Multiple solid tumors	Phase I/II	Recruiting
	NCT05701553	S-adenosyl-methionine (SAM) and anti-PD-1/PD-L1 antibodies	Methionine metabolism	Hepatocellular carcinoma	Observational	Recruiting

## Abbreviations

5FU	5-fluorouracil
αKG	Alpha-ketoglutarate
ACC-1	Acetyl-CoA carboxylase-1
AHCY	Adenosylhomocysteinase
AhR	Aryl hydrocarbon receptor
ARG-1	Arginase 1
ASS1	Argininosuccinate synthetase 1
BCAA	Branched-chain amino acids
CAF	Cancer-associated fibroblasts
CD204	Scavenger receptor A
CRC	Colorectal cancer
CSF3	Colony stimulating factor 3
DC	Tumor resident dendritic cells
DHAP	Dihydroxyacetone phosphate
DON	6-diazo-5-oxo-norleucine
ETC	Electron transport chain
EAA	Essential amino acid
EMT	Epithelial to mesenchymal transition
EV	Extracellular vesicle
FAS	Fatty acid synthesis
FAO	Fatty acid oxidation
FAS	Fatty acid synthesis
FoxP3	Forkhead box P3
G3P	Glyceraldehyde-3-phosphate
GLS	Glutaminase
GLUT1	Glucose transporter
GSH	Glutathione
HIF-1α/2α/3α, HIF-1β	Hypoxia inducible factors
HK	Hexokinase
IDH	Isocitrate dehydrogenase
IDO	Indoleamine 2,3-dioxygenase
IFNγ	Interferon-γ
IRG1	Cis-aconitate decarboxylase
LAP	Laryngeal adductor paralysis
LDHA/LDHB/LDH	Lactate dehydrogenase
MAT2A	Methionine adenosyltransferase 2A
MCT/MCT1/MCT4	Monocarboxylate transporters
MDSC	Myeloid-derived suppressor cells
MYC	MYC proto-oncogene
NEAA	Non-essential amino acid
NFAT	Nuclear factor of activated T cells
NK	Natural killer cell
NO	Nitric Oxide
NOS	Nitric oxide synthase
NSCLC	Non-small cell lung cancer
OXPHOS	Oxidative Phosphorylation
PDAC	Pancreatic adenocarcinoma
PD-1	Programmed cell death protein 1
PD-L1	Programmed death-ligand 1
PDH	Pyruvate dehydrogenase
PDHK1	Pyruvate dehydrogenase kinase
PFK	Phosphofructokinase
PGE_2_	Prostaglandin E2
PHD	Prolyl-4-hydroxylase
PI3K	Phosphoinositide 3-kinase
PPAR	Peroxisome proliferator-activated receptors
PPP	Pentose phosphate pathway
ROS	Reactive oxygen species
SAH	S-adenosylhomocysteine
SAM	S-adenosylmethionine
SDH	Succinate dehydrogenase
SREBP	Sterol regulatory element binding proteins
SUCLA2	Succinyl CoA synthetase
SUCNR1	Succinate receptor 1
TAM	Tumor-associated macrophages
TCA	Tricarboxylic acid cycle
TCR	T cell receptor
TDE	Tumor-derived exosome
Teff	Effector T cells
TET	Ten-eleven translocase
TIL	Tumor-infiltrating lymphocytes
TLR	Toll-like receptor
TME	Tumor microenvironment
Tmem	Memory T cells
Treg	Regulatory T cells
UPR	Unfolded protein response
VEGF	Vascular endothelial growth factor
